# Avatar diversity perception scale (ADPS): a new multidimensional measure for perceived human avatar diversity

**DOI:** 10.3389/fpsyg.2025.1705591

**Published:** 2025-11-11

**Authors:** Xiangting Bernice Lin, Chen Lou, Moon-Ho Ringo Ho

**Affiliations:** Nanyang Technological University, Singapore, Singapore

**Keywords:** perceived diversity, avatar, scale development, construct validity, reliability

## Abstract

**Introduction:**

Avatars are increasingly used as digital representations of human diversity. However, the study of how avatar diversity is perceived is complicated by significant conceptual challenges. Thus, this research seeks to engage in early theory building by developing a measure of perceived avatar diversity.

**Methods:**

Through preliminary qualitative interviews and a survey, study 1 establishes four dimensions of the perceived diversity. Study 2 provides initial validation for the factor structure via a confirmatory factor analysis.

**Results:**

Following analyses and model adjustments, the studies support a 23-item, three-dimensional structure, consisting of perceived heterogeneity (variation in avatars), diversity concerns (adverse reactions to using avatars for diversity), and context-dependent diversity (time- and community-bound nature of avatar diversity).

**Discussion:**

This scale development lays a foundation for future work investigating the antecedents and mechanisms accounting for perceived avatar diversity. Our findings offer industry practitioners actionable principles in creating avatars that are heterogenous, authentic, relatable, and dynamic for effective diversity representations.

## 1 Introduction

Fueled by advancements in technological infrastructure, the digital human market is predicted to reach between $125 billion and $440.3 billion by the early 2030s (Jiang, 2023). This includes human avatars, which represent actual human users or nonhuman entities and are becoming increasingly lifelike due to cutting-edge 3D modeling technology (Bailenson and Beall, 2006; Burden and Savin-Baden, 2019; Miao et al., 2022). Examples include customizable avatars developed by Meta for platform users, and virtual influencers created and controlled by software or artificial intelligence (AI) for advertising and marketing (e.g., Lil Miquela, Shudu). Because such avatars are employed by users to mediate online interactions and by commercial entities to personify themselves and access their target audience, there is mounting interest for avatars to reflect the diversity of the people they represent and target (Do et al., 2023).

Human diversity can be described as the differences between individuals on any attribute that lead to the perception of another as different (Daniels et al., 2017; Homan et al., 2010; van Knippenberg et al., 2004; van Knippenberg et al., 2007; Williams and O’Reilly, 1998). In the absence of a unified theory on avatar diversity, many studies directly apply principles of human diversity to the study of avatars. These studies have explored user responses to specific human attributes, primarily focusing on avatar race, gender, and age (Hong et al., 2024; Lee, 2014; Lee et al., 2018; Lee and Yuan, 2023; Lehdonvirta et al., 2012; Yang et al., 2014; Zhang et al., 2020; Zhang et al., 2017). They are premised on different social perception or communication media perspectives: for example, Lee and Yuan (2023) and Yang et al. (2014) formulate their predictions about avatar gender and race effects, respectively, based on stereotype activation. Lehdonvirta et al. (2012) considers gender effects based on social role and expectations. Others rely on an eclectic combination of concepts, including social role, stereotyping, social presence, media richness, and evolutionary theory (Zhang et al., 2020; Zhang et al., 2017). Still others do not reference any theory, instead formulating predictions based on existing empirical findings (Ferraro et al., 2024; Lee et al., 2018). While essential for understanding specific dimensions of avatar diversity, a key problem with the present approaches is that they are predominantly one-dimensional and identity-based, rather than diversity-centric. This study addresses a gap by proposing a diversity-centric theory of perceived avatar diversity that integrates principles from social perception and the specific characteristics of avatars as a communication medium.

Recognizing that a diversity-centric framework treats diversity as fundamentally multidimensional, the foundational step in constructing such a theory is to identify its core components. Thus, this study is guided by the research question: *Which factors constitute the perception of avatar diversity?* We leverage theories and research on social identity, person perception, impression management, and the uncanny valley effect (Goffman, 1959; Mori, 1970; Tajfel et al., 1979; Tajfel and Turner, 2004) and seek to test five proposed components of perceived avatar diversity, namely, *perceived heterogeneity*, *salience*, *sense of representation*, *representation fidelity,* and *context-dependent diversity*. We conducted two studies: study 1 developed the model using qualitative interviews, a survey, and exploratory factor analysis (EFA) and study 2 provided initial validation for its dimensions with confirmatory factor analysis (CFA). Our studies contribute to early theory building and provide a three-dimensional framework of perceived avatar diversity, comprising its perceived heterogeneity, diversity concerns, and context-dependent diversity.

The current study fills a theoretical void by establishing an initial diversity-centric, multidimensional model of perceived avatar diversity, as well as a means to measure it. In doing so, we directly connect human diversity theories to avatar diversity. We also reconceptualize perceived avatar diversity by shifting the focus from mirroring the infinite number of human attributes to the multidimensionality that emerges at the intersection of social perception and avatars’ unique properties. Since adverse reactions to avatar diversity are also shown to be driven by design and representation failures, rather than an us-vs.-them divide and intergroup bias, we demonstrate that theories integrating social perception with communication media perspectives provide a more robust explanation than either one can offer in isolation. The present findings offer actionable insights for industry practitioners, specifically creating avatars that are heterogenous, authentic, relatable, and dynamic for effective diversity representations.

## 2 Literature review

### 2.1 Perceived human diversity

Perceived diversity is a multifaceted construct in human diversity research, viewed variably through its functional role (e.g., as a mediator) and its structural form (e.g., as subgroup splits or heterogeneity; Shemla et al., 2016; Shemla et al., 2024). Shemla et al. (2016) defines perceived diversity as the “degree to which members are aware of one another’s differences” (p. S91). Despite its widespread study, the construct remains problematic, including its frequent conflation with objective diversity. This conflation assumes that perceived differences are simply a direct awareness of the actual differences that are present (Shemla et al., 2024). Yet, empirical evidence contradicts this, showing that perceived diversity is often biased, inaccurate, and influenced by the perceiver’s social goals or focal points of research (Daniels et al., 2017; Shemla et al., 2016; Unzueta et al., 2012), thereby driving a disconnect between theory on objective diversity and measurement that is based on perceived diversity. To address the gap, this paper explicitly sets out to develop a model of avatar diversity from the users’ perspective.

Another fundamental disconnect impedes progress in diversity research, where perceived diversity oscillates between a narrowly fixed list of one or more attribute(s) in practice and an excessively broad range of individual differences in principle. Much of the literature operationalizes human diversity by focusing on one or a few selected attribute(s), such as age, race, gender, and social class (Connor et al., 2022; Daniels et al., 2017; Jaffé et al., 2019; Kauff et al., 2019; Petsko et al., 2022; Starck et al., 2021). This approach risks conceptual tautology. For example, if “perceived racial diversity” is simply the perception of race, then it becomes difficult to argue that “perceived diversity” is a distinct construct, offering little new explanatory power beyond the established study of racial attitudes or stereotypes. In fact, the concept of perceived diversity is theoretically unbounded, as it can be informed by a limitless array of individual differences (Daniels et al., 2017; Homan et al., 2010; van Knippenberg et al., 2004; van Knippenberg et al., 2007; Williams and O’Reilly, 1998). This creates a crisis in measurement. Since any two individuals differ in infinite dimensions, even beyond what is visible (Harrison et al., 2002; Phillips and Loyd, 2006; Phillips et al., 2006), the concept becomes so expansive as to be analytically imprecise. If any perceived difference constitutes diversity, perceived diversity loses its theoretical specificity and utility.

### 2.2 Perceived avatar diversity

The foregoing conceptual issues are further complicated because existing theories of human diversity were not designed to address the unique complexities of the digital realm. To date, efforts to clarify the perceived human diversity construct are domain-specific and an aggregation of empirical research on myriad sociodemographic classifications driven by different theoretical paradigms (e.g., social identity/ self-categorization, intergroup threat, categorization-elaboration model; Campbell et al., 2025; van Knippenberg et al., 2004). While this body of work is foundational for diversity research, its application to the digital realm is not straightforward, lacking the specific coherence needed for meaningful predictions in a dynamic context shaped by factors like user-controlled avatar design and algorithmic content creation or exposure. Users can alter how they look without adhering to their actual identities and appearances (Walther, 2007; Walther and Lew, 2022) or without even noticing diverse others, given asynchronous interactions and the vast and often distracting array of online content. New diverse human avatars can also be introduced with generative AI or AI-based recommendation systems in promoting diversity. The current study disentangles avatar diversity from human diversity in conceptualizing how the former is perceived, particularly by accounting for its “material” characteristics.

Amidst a jarring theoretical void on avatar diversity, few studies have examined perceived avatar diversity as an independent, standalone construct. Studies alluding explicitly to avatar diversity are recent and explore the limitations of current technologies in supporting diversity representations (Morgan et al., 2020) or whether and when diverse individuals would disclose relevant markers through avatars (Lee, 2014; Mack et al., 2023; Zhang et al., 2022), mostly using qualitative approaches. Others shift away from users’ self-presentations to examine consumer reactions to diverse avatars, such as in digital advertising contexts (Ferraro et al., 2024; Sands et al., 2024). These studies replicate the conceptual issues in human diversity research by focusing on a single attribute and drawing on an eclectic range of theories and concepts, including self-presentation/ impression management (Zhang et al., 2022), social identity/ self-categorization (Lee, 2014; Sands et al., 2024), and the social model of disability (Mack et al., 2023). A critical limitation is the unexamined presumptions of an equivalence between humans and avatars and a direct translation of perceptions of human diversity to that of avatar diversity, thereby neglecting the unique psychological processes involved in interpreting digital representations. Recent scholarship (Shemla et al., 2024) rightly calls for establishing perceived avatar diversity as a unique psychological construct, underscoring the need to investigate how users truly perceive avatar diversity. Whether and how users perceive avatar diversity cannot be assumed to be a direct reflection of the objective human diversity, if measurable at all.

Consequently, this study seeks to initiate a new, multidimensional model of perceived avatar diversity by shifting away from the theoretically boundless list of attribute differences and integrating research on human and avatar diversity. Specifically, we turn our focus to the social perception and unique characteristics of human avatars and posit five dimensions of perceived avatar diversity, including (1) *perceived heterogeneity*, (2) *salience*, (3) *sense of representation*, (4) *representation fidelity*, and (5) *context-dependent nature*. The following sections address each dimension in greater detail.

### 2.3 Perceived heterogeneity

The perception of diversity among human avatars is fundamentally shaped by their heterogeneity, i.e., the degree of variation or differences present in human avatars. While diversity in human teams is a double-edged sword (van Knippenberg et al., 2004), research shows that the framing of diversity is key. Perceptions focused on subgroup splits or self-to-team dissimilarity evoke a divisive “us-vs.-them” mentality and adverse reactions (Shemla et al., 2016; van Knippenberg et al., 2004), processes that have been well-established by social identity/ self-categorization theory (Tajfel et al., 1979; Tajfel and Turner, 2004). In contrast, diversity perceived abstractly and through the lens of heterogeneity produces positive outcomes (Shemla et al., 2016; Toma et al., 2025). Responses to diverse avatars have been predominantly positive (Ferraro et al., 2024; Lee, 2014). Because human avatars are inherently abstractions, and unless certain avatar features are intentionally made salient, their diversity is best conceived in terms of heterogeneity.

The heterogeneity of avatars acknowledges not only arbitrary social categorizations but also the rich network of continuous differences among them. This is amplified by the technology used to create human avatars. Unlike in human interaction where features form a holistic identity, technologies like avatar customization tools and generative AI deconstruct appearance into granular attributes. Users and creators directly choose, manipulate, or prompt for specific traits like eye shape and hair style (Messinger et al., 2019). Such processes elevate the importance of feature-level differences, rendering perceived heterogeneity the dominant lens for assessing diversity in virtual spaces. Therefore, our understanding of avatar diversity spans from broad categorical labels (Tajfel et al., 1979; Tajfel and Turner, 2004) to the specific, distinct features that define each individual avatar.

### 2.4 Salience

The dimension of salience is the extent to which the diversity of human avatars stands out to the perceiver. Since diversity is not always at the forefront of one’s mind (Mayo et al., 2016), its perception is not a given. Person perception and social identity research suggest that individuals attend to differences that are contextually primed, such as minority status (Abrams et al., 1990; Randel, 2002), deviations from mental prototypes (Cohen, 1981), or specific social motivations (Unzueta et al., 2012).

This principle of salience is especially critical for human avatars, whose diversity is, by definition, surface-level (e.g., appearance). Surface-level attributes are readily observable or visible, while deep-level attributes emerge through interaction (Harrison et al., 1998; Harrison et al., 2002; Meyer et al., 2011; Phillips and Loyd, 2006; Phillips et al., 2006). Although digital platforms can easily render surface-level diversity, individuals consider deep-level attributes more significant when asked what differences stand out most to them (Meyer et al., 2011). This creates a fundamental challenge, where the aspects of diversity that may be most noticeable to users are often the least immediately visible in a human avatar representation. Therefore, the dimension of salience is essential, as it captures the gap between the existence of diversity and its perception.

### 2.5 Sense of representation

The sense of representation captures the degree to which users feel that everyone is effectively represented by the prevailing set of human avatars. People want to be included, and individuals relate to diverse representations well depending on the extent to which their identities and experiences, and others close to them, are sufficiently represented (Burgess et al., 2024; Burgess et al., 2021). At its core, this sense is rooted in the fundamental human need for belonging (Baumeister and Leary, 2017) and self-presentation/ impression management (Goffman, 1959). Indeed, users are motivated to manage how they are perceived by others in virtual spaces (Walther, 2007). While peripheral features might be adjusted, people continue to ensure a close alignment between one’s actual and digital identities (Messinger et al., 2019), which in turn evoke stronger psychological connections (Waltemate et al., 2018). Besides, users are disenchanted by a prevailing lack of inclusive options in avatar customization (Morgan et al., 2020; Zhang et al., 2022) and self-disclosure of one’s diverse identities are more likely with greater objective diversity (Lee, 2014). Broadly then, perceiving oneself to be represented is a key element of a positive diversity experience.

A robust sense of representation extends beyond the self to include the perception that others are also represented. Users generally welcome a wide array of avatar customization options and virtual humans (Ferraro et al., 2024; Morgan et al., 2020), reflecting an appreciation for a visibly diverse virtual environment. However, the matter is more nuanced, as favorable perceptions of having diverse others by the majority are attributed to novelty and instrumental beliefs about diversity (Ferraro et al., 2024; Homan et al., 2010; van Knippenberg et al., 2007), motivations that are perceived as alienating by the minority (Starck et al., 2021). Additionally, the salience of certain diversity markers can elicit negative reactions, such as discrimination rooted in ingroup bias, perceived threat, or lack of empathy (Iyer, 2022; Lindsey et al., 2015; Stephan et al., 2015; Tajfel et al., 1979; Tajfel and Turner, 2004; van Knippenberg et al., 2004). From this view, “good” objective representation can become subjectively narrow and exclusive, lacking the crucial elements of empathy, perspective-taking, and interpersonal sensitivities that underpin a strong sense of inclusion. Accordingly, senses of representation entail feelings of both self and others to be adequately represented, and the dimension measures whether a virtual space successfully bridges personal identification with an empathetic recognition of others.

### 2.6 Representation fidelity

A closely related dimension to the felt sense of representation is representation fidelity, which is the degree to which digital representations are assessed to be accurate and authentic portrayals of actual human diversity. Fidelity is influenced by the affordances of technological tools and user or creators’ behavior. People often prefer digital selves that are consistent with their actual appearance (Messinger et al., 2019) and find more realistic avatars to be cooler and more credible (Kim et al., 2024; Nowak and Rauh, 2005). In theory, higher fidelity should enable a more faithful representation of human diversity by capturing its nuances in detail. However, this relationship is complicated by two factors, demonstrating that fidelity cannot be measured by technical realism alone. First, the uncanny valley theory warns that excessive realism can trigger repulsion, undermining the very connection it seeks to build (Mori, 1970). Human avatars exist on a spectrum from cartoonish to humanlike (Nowak, 2004; Zhang et al., 2022), and the uncanny valley reveals that simply maximizing realism can be counterproductive. This suggests that effective representation requires a calibrated realism, one that is assessed to be sufficiently accurate without becoming unsettling.

Second, high fidelity can be compromised by a lack of authenticity. High-fidelity human avatars built on narrow, preconceived notions risk creating and amplifying harmful stereotypes rather than genuine representations. People react poorly to diversity representation that is perceived to lack authenticity (Campbell et al., 2025). Members of underrepresented groups respond poorly to diverse virtual humans, preferring representation by real humans instead (Sands et al., 2024). A large body of research further confirms that stereotypical media portrayals inflict social and psychological harms, such as marginalization, violence, and poor physical and mental well-being (Appel and Weber, 2021; Campbell et al., 2025; Ramasubramanian et al., 2023; Santoniccolo et al., 2023). In fact, accurate, nuanced, and respectful portrayals are advocated as a powerful tool to challenge stereotypes and reduce prejudice (Eisend et al., 2023; Ramasubramanian et al., 2023). Thus, diverse representations are about not only the flawless rendering of pixels, but also the faithful depictions of people.

### 2.7 Context-dependent diversity

An emerging conception of diversity relates to the perceiver’s awareness of diversity’s evolving and circumstantial nature (Shemla et al., 2024). Diversity is bound by community, time, and space. What is considered representatively diverse in one’s perceived community is not in another’s. Diversity also changes with time: for instance, previously less visible attribute differences, such as LGBTQIA+ and disabilities, have become more apparent and resulted in legislative changes over the course of American history (Tessema et al., 2023). New social movements fueled by the media (e.g., #BlackLivesMatter) have emerged in more recent times (Kratz, 2024). Further, the definition and relative importance placed on different aspects of diversity vary across cultures. Race is often the center of diversity discourse in the United States, gender in East Asian cultures, and people’s characterizations of diversity are amorphous (Unzueta et al., 2012). Extended to virtual spaces, perceptions of avatar diversity recognize that diversity is situated and dynamic, especially as users come and go, online interactions and cultures shift, platforms vary, and technologies change.

## 3 Study 1: item generation and model testing

### 3.1 Item generation

Potential scale items were generated through one-on-one semi-structured interviews with a diverse group of participants. A total of 26 participants (male = 13, female = 13; *M_age_* = 37.4, *SD_age_* = 14.0) with at least a moderate level of experience with human avatars were included and interviewed. Each interview lasted about an hour. All participants were compensated about US$19.50 for completing the interview. Study procedures have been approved by the university’s Institutional Review Board (reference code: IRB-2025-025). Informed consent was obtained prior to study commencement.

Items from the audio transcriptions that reflect our various proposed dimensions of perceived avatar diversity were labeled. A total of 712 items were generated verbatim by reviewing the face validity of items and item alignment with the theoretical dimensions. Items that contained jargon or repeated meanings were removed, and 65 items were finalized for EFA (Appendix A). Minor wording refinements were made to these items to improve clarity. All items used a 7-point Likert scale ranging from 1 (strongly disagree) to 7 (strongly agree).

### 3.2 Sample and procedure

Participants aged between 18 and 75 years old with at least a moderate level of experience with human avatars from the United States were recruited through Prolific to complete the anonymized survey on Qualtrics. Study participation took about 10 min. All participants received about US$2.00 upon survey completion. A total of 465 individuals were analyzed using EFA. The mean age was 40.4 years old (*SD* = 13.0). Majority were male (*N* = 237, 51.0%) and the rest were female (*N* = 218, 46.9%), were transgender (*N* = 8, 1.7%), or prefer not to say (*N* = 2, 0.4%). Most were White/ Caucasian (*N* = 315, 67.7%) and the remaining were Black/ African American (*N* = 129, 27.7%), Asian (*N* = 22, 4.8%), American Indian/ Alaska Native (*N* = 6, 1.2%), or Other (*N* = 8, 1.7%).

### 3.3 Exploratory factor analysis

Kaiser-Meyer-Olkin (KMO) and Bartlett’s test of sphericity were conducted. Items with a KMO value of at least 0.80 were indicative of sample adequacy, and a *p*-value of < 0.05 for Bartlett’s test indicated data suitability by significant correlations among the underlying factors (Hair et al., 2019, p. 136). Kaiser’s eigenvalue >1 criterion and Horn’s parallel analysis (PA) using principal axis factor analysis at 99% and 5,000 resamples were used to determine the number of factors.

Principal axis factoring followed by the Promax method for oblique rotation were implemented to identify the underlying unobservable latent factors for parsimony, while explaining a large amount of common variance of the directly observable variables or items. To ensure that the resulting factor structure was theoretically sound, items with very low communalities (< 0.20) were first removed (Child, 2006). Then, items that were theoretically irrelevant to the underlying factors or loaded onto multiple factors (cross-loadings > 0.40) were removed systematically (Hair et al., 2019, p. 151). EFA was conducted again with every adjustment of an item. Rotated factor loadings for items greater than 0.40 in the final EFA were reported.

## 4 Study 1 results

The KMO value was 0.89 and the Bartlett’s test was *p* < 0.001, indicating sample adequacy and data suitability for factor analysis. The initial EFA based on both PA and Kaiser’s criterion suggested a four-factor solution, contrary to the five theorized dimensions. Closer examination of the rotated factor loadings revealed that the factors that items loaded onto did not align completely with our proposed dimensions of diversity perception. Specifically, five items did not load onto the proposed “perceived heterogeneity,” and two items under the proposed “salience” loaded onto the same factor as the other eight “perceived heterogeneity” items. Only four other items from “salience” loaded variably across two other factors, suggesting that “salience” may not be significant to the self-rated measurement of diversity perception.

Separately, six items aligned with our proposed dimension of “context-dependent diversity,” while four loaded variably across two other factors. Two additional items failed to align with their respective dimensions and loaded onto “context-dependent diversity” instead. Regarding our proposed dimension of “representation fidelity,” five items aligned and loaded onto the same factor; seven more items from other dimensions also loaded with these five items. Closer examination reveals perceivers’ intrinsic care for accurate representations beyond the mere verification of fidelity that was originally conceptualized. Thus, the dimension relates more precisely to a “drive for fidelity.” Finally, only four items aligned with our proposed “sense of representation.” Thirteen additional items loaded onto this same factor. Closer inspection indicates that these items constitute a new dimension of “diversity concerns” that is characterized by apathy, dissatisfaction, doubt, or discomfort with representing human diversity using human avatars.

Nine items with very low communalities were removed one at a time. The content of all items and their corresponding factors were then reviewed systematically. A total of 7 items that were theoretically irrelevant were deleted in sequence. Nine items with low loadings were eliminated. Following this, 40 items loaded onto four distinct factors, which were corroborated by PA and Kaiser’s criterion. The four-factor solution accounted for 36.2% of the total variance, with the first factor explaining 16.5% of the total variance. Fourteen items loaded onto diversity concerns, 10 onto drive for fidelity, 9 onto perceived heterogeneity, and 7 onto context-dependent diversity. All the items demonstrated moderate to high factor loadings (> 0.40) on their respective factors (Table 1).

**Table 1:** Rotated factor loadings (*N* = 465).

Item	Four-factor solution (40 items)			
	1: Diversity concerns	2: Drive for fidelity	3: Perceived heterogeneity	4: Context-dependent diversity
14	0.57			
22	0.56			
27	0.59			
34	0.68			
36	0.52			
38	0.61			
40	0.53			
41	0.42			
44	0.53			
45	0.55			
50	0.49			
52	0.45			
58	0.52			
60	0.58			
9		0.48		
15		0.50		
30		0.55		
31		0.48		
33		0.49		
39		0.60		
42		0.43		
43		0.51		
46		0.44		
51		0.52		
1			0.49	
2			0.45	
3			0.41	
5			0.62	
7			0.66	
8			0.44	
12			0.55	
13			0.45	
18			0.77	
37				0.56
54				0.43
55				0.42
56				0.61
57				0.73
59				0.43
65				0.41
Unadjusted eigenvalue	7.20	5.52	2.54	1.70
% of variance before rotation	16.51	12.16	4.77	2.75

Extraction method: principal axis factoring; rotation method: oblique Promax. Loading values < 0.40 were omitted.

Finally, the four dimensions of the perception of avatar diversity were refined for clarity. *Diversity concerns* describe the adverse reactions, such as general apathy, dissatisfaction, doubt, or discomfort, that users have regarding diverse representations with human avatars. *Drive for fidelity* is the degree to which users seek and value accurate and authentic portrayals of diversity. *Perceived heterogeneity* refers to the overt recognition of the variation or differences among human avatars. Last, *context-dependent diversity* reflects the degree to which avatar diversity is defined by time and the actual community of users.

## 5 Study 2: model validation

After determining the factor structure, study 2 sought to validate the model with an independent sample. Initial model validation was conducted by assessing model fit, convergent validity, discriminant validity, and internal consistency reliability of the avatar diversity perception scale (ADPS) full and subscales.

### 5.1 Sample and procedure

Study enrollment procedures were identical to study 1. A total of 417 individuals were analyzed using CFA. The mean age was 36.5 years old (*SD* = 12.1). Majority were female (*N* = 232, 55.6%) and the rest were male (*N* = 177, 42.4%), were transgender (*N* = 5, 1.2%), or prefer not to say (*N* = 3, 0.7%). Most were White/ Caucasian (*N* = 274, 65.7%) and the remaining were Black/ African American (*N* = 130, 31.2%), Asian (*N* = 9, 2.1%), American Indian/ Alaska Native (*N* = 2, 0.4%), or Other (*N* = 9, 2.1%).

### 5.2 Confirmatory factor analysis

CFA with maximum likelihood estimation was used to verify the factor structure derived from EFA with an independent sample. Items that loaded on the same factor in the EFA were allowed to load onto the corresponding latent factor in the CFA. The variance of the latent factor was fixed to 1 and all factor loadings for that latent factor were then freely estimated. Standardized factor loadings were generated for each item within the latent factors and items with loadings < 0.50 were deleted. Model fit was assessed using the following fit statistics and criteria: chi-square to degrees of freedom ratio (*χ^2^/df*) of 3 or lower, Comparative Fit Index (CFI) and Tucker-Lewis Fit Index (TLI) of 0.90 or higher, Root Mean Square Error of Approximation (RMSEA) of 0.06 or lower, and Standardized Root Mean Square Residual (SRMR) of 0.08 or lower (Ge et al., 2025; Hair et al., 2019).

### 5.3 Construct validity

The convergent and discriminant validity of the ADPS were examined. Convergent validity was evaluated by assessing the correlations between the ADPS dimensions and known adapted measures of perceived workgroup diversity (Fellnhofer et al., 2017), pro-diversity beliefs (Homan et al., 2010; Homan et al., 2007), and openness to experience (Ashton and Lee, 2009). Discriminant validity was evaluated by assessing the correlations between the ADPS dimensions and measures of avatar technology acceptance (Bangor et al., 2008; Brooke, 1996) and extraversion (Ashton and Lee, 2009). Pearson product–moment correlation coefficients *r* ≥ 0.40 indicate at least moderate correlations or convergent validity, and *r* < 0.40 indicate weak correlations or discriminant validity between the ADPS dimensions and the validity measures (Schober et al., 2018). Studies suggest that stronger pro-diversity beliefs predicted increased likelihoods of perceiving diversity, and personality traits of openness to experience significantly associated with pro-diversity attitudes whereas extraversion had no such association (Han and Pistole, 2017; Jaffé et al., 2022).

Scale internal consistency reliability was also assessed. The internal consistency of each dimension and the entire ADPS were evaluated in both EFA and CFA datasets. Cronbach’s alpha values of 0.70 or higher were deemed acceptable, suggesting that the items within each subscale and the full ADPS consistently measure the same construct(s).

## 6 Study 2: results

### 6.1 Model fit

**Figure 1:**
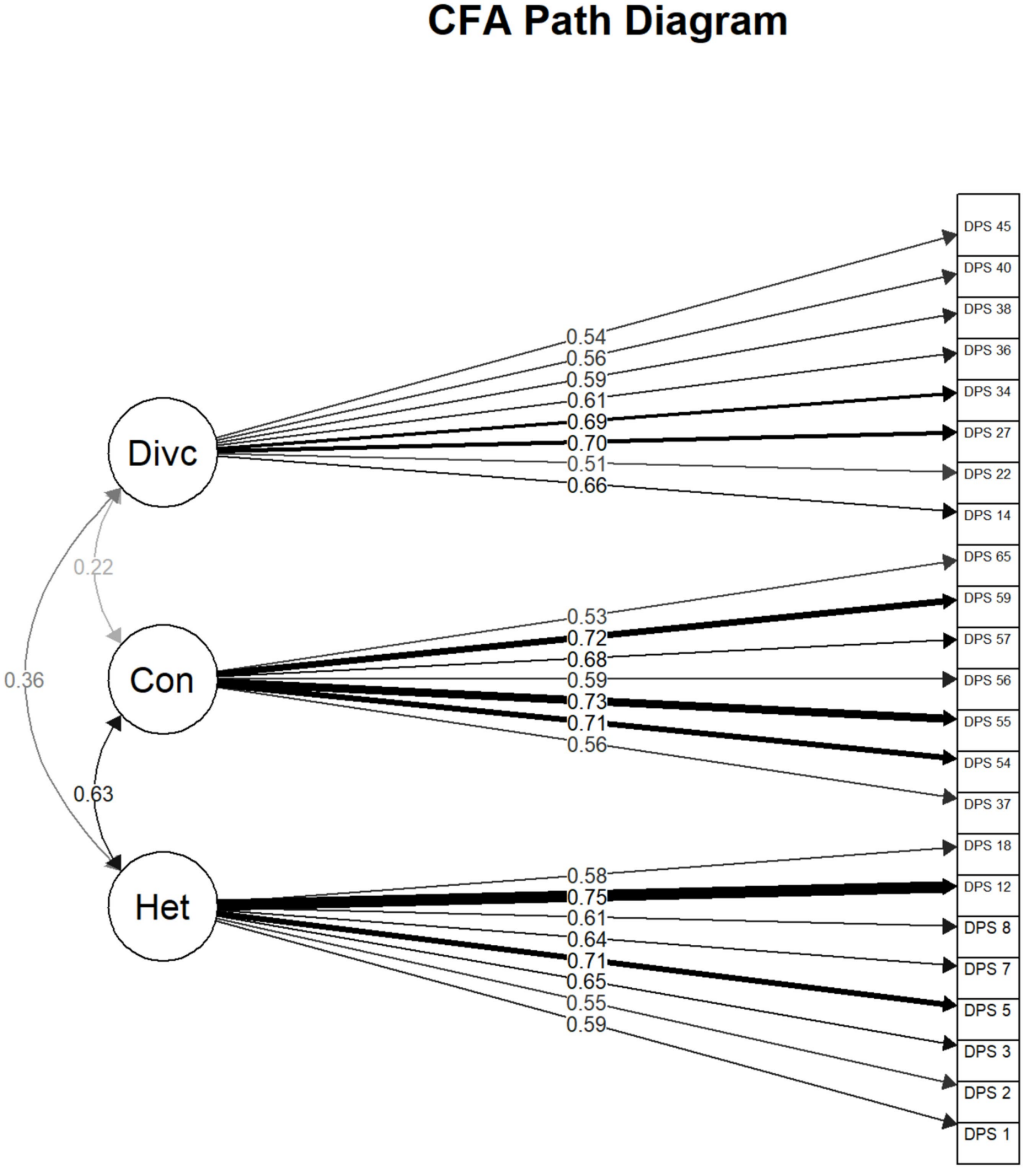
Confirmatory factor analysis of a three-factor model. Divc, diversity concerns; con, context-dependent diversity; het, perceived heterogeneity. This figure was generated using R (version 2025.05.0) and the semPlot package (version 1.1.6).

Fifteen items demonstrated low factor loadings < 0.50 and were systematically removed. Most of these deleted items constituted the dimension of drive for fidelity, suggesting that the factor could be problematic. CFA was re-conducted with the remaining 25 items with acceptable factor loadings based on the four-factor structure identified in the EFA. The *χ^2^/df* was 2.24 (*χ^2^* (269) = 601.56, *p* < 0.001), CFI = 0.91, TLI = 0.90, RMSEA 0.05, 90% CI [0.05, 0.06], and SRMR = 0.06. These goodness-of-fit indices indicated that the four-factor model with 25 items was acceptable. However, the dimension of drive for fidelity comprising of two items showed high correlations (> 0.70) with perceived heterogeneity and context-dependent diversity, indicating poor discriminant validity. The model did not converge when a higher order factor was modeled for the correlated factors. Evidence continued to suggest model destabilization due to a two-item drive for fidelity. After removing this factor, the factor structure was stable and fit indices improved, *χ^2^/df* = 2.17 (*χ^2^* (227) = 493.13, *p* < 0.001), CFI = 0.92, TLI = 0.91, RMSEA 0.05, 90% CI [0.05, 0.06], and SRMR = 0.06. Altogether, the CFA dataset supports a 23-item, three-factor model of diversity perception. The final factor structure departed from our initial expectations, and CFA required several adjustments based on modification indices to achieve adequate model fit, which is common to early-stage scale development. The standardized factor loadings for each item on these respective latent variables are shown in Figure 1.

### 6.2 Convergent validity

Pearson product–moment correlation coefficients indicated variability in correlations between the ADPS dimensions and known measures of perceived diversity, pro-diversity beliefs, and openness to experience. Specifically, perceived heterogeneity (*r* = 0.46) and context-dependent diversity (*r* = 0.42) were moderately correlated with the adapted measure of perceived diversity, reflecting convergent validity. Context-dependent diversity was also moderately correlated with pro-diversity beliefs (*r* = 0.62). Diversity concerns (*r* = 0.41) and context-dependent diversity (*r* = 0.43) were moderately correlated with openness to experience. The remaining correlations were relatively weaker, *r* < 0.40.

### 6.3 Discriminant validity

Likewise, correlations between the ADPS dimensions and measures of avatar technology acceptance and extraversion were variable. Context-dependent diversity was weakly correlated with both technology acceptance (*r* = 0.23) and extraversion (*r* = 0.25), indicating discriminant validity. Perceived heterogeneity (*r* = 0.36) and diversity concerns (*r* = 0.30) were weakly correlated with extraversion. The remaining correlations were relatively moderately stronger, *r* ≥ 0.40. Table 2 summarizes the correlations between the ADPS dimensions and validity measures.

**Table 2:** Correlations between ADPS dimensions and validity measures (Pearson’s r).

ADPS dimension	Convergent validity			Discriminant validity	
	Perceived diversity	Pro-diversity beliefs	Openness to experience	Avatar technology acceptance	Extraversion
Diversity concerns	0.23	0.28	0.41	0.59	0.30
Perceived heterogeneity	0.46	0.36	0.33	0.45	0.36
Context-dependent diversity	0.42	0.62	0.43	0.23	0.25

All Pearson’s r were significant at *p-*value < 0.01.

### 6.4 Internal consistency reliability

The internal consistency reliability of the 23-item ADPS for both EFA and CFA datasets were acceptable. Cronbach’s alpha of the ADPS full and subscales ranged from 0.78 to 0.84. Table 3 provides the item descriptions for the 23-item ADPS and summarizes these findings.

**Table 3:** Scale reliability.

Dimension	Item	Cronbach’s alpha (EFA)	Cronbach’s alpha (CFA)
Diversity concerns	14* I do not really notice the diversity.	0.80	0.82
	22* It is hard to spot distinct differences.		
	27* I feel like I am not represented.		
	34* I cannot even relate to what is represented.		
	36* I am not the target audience.		
	38* The diversity makes people feel uncomfortable.		
	40* The avatars do not even look human.		
	45* I question if the diversity is real.		
Perceived heterogeneity	1 There is variation in general.	0.84	0.84
	2 There is difference in the looks.		
	3 There are different expressions on the face.		
	5 There is representation of various groups, identities, and perspectives.		
	7 There is a good representation of various interest groups and communities.		
	8 There are different skin colors, facial features, hair, body shapes and physical disabilities.		
	12 There is a range of different features.		
	18 It is obvious that there is already diversity.		
Context-dependent diversity	37 The people that are creating avatars need to be diverse in order for avatars to be diverse.	0.78	0.83
	54 Diversity is work in progress.		
	55 Diversity can be achieved over time.		
	56 Diversity is anything that is rooted in the community.		
	57 Diversity is anything that shapes the community.		
	59 Diversity is evolving all the time.		
	65 Society is still trying to accept this.		
23-item ADPS		0.82	0.86

Items with an (*) are reverse-coded.

## 7 Discussion

This exploratory research seeks to contribute to early theory building by developing a measure of perceived avatar diversity. Broadly, we found support for our initial dimensions of perceived heterogeneity and context-dependent diversity. We did not find consistent support for the dimensions of salience or representation fidelity. While the sense of representation was intended to capture both positive and negative affective responses to avatar diversity, the prevailing sense is better marked by apathy, dissatisfaction, doubt, or discomfort with using avatars to represent diversity.

To the authors’ knowledge, there is neither a diversity-centric theory nor a well-established scale on perceived avatar diversity. Our findings provide a preliminary multidimensional measure of perceived avatar diversity, beyond listing the infinite dimensions on which individuals differ (Homan et al., 2010; Kauff et al., 2019; van Knippenberg et al., 2004; Williams and O’Reilly, 1998). We contest the presumption of perceived diversity as equivalent to the objective differences (Shemla et al., 2024) and the direct application of human diversity theories to avatar diversity (Lee, 2014; Mack et al., 2023; Zhang et al., 2022). Critically, we demonstrate that people recognize the variation across avatars but continue to have reservations about using them to represent human diversity, thereby concurring with research showing how people prefer human representations to digitally created substitutes (Sands et al., 2024). Finally, we offer a direct response to Shemla et al. (2024) by presenting the first empirically-driven measure of the dynamic nature of diversity.

### 7.1 Theoretical implications

Our study makes several theoretical contributions. First, we advance diversity research with our novel conceptual model that is specific to the human avatar diversity. Study findings show that users perceive avatar diversity in terms of its heterogeneity, thereby integrating theorizations on how the perceived heterogeneity of workgroups produces positive diversity outcomes (Shemla et al., 2016) and emerging research demonstrating the favorable effects of avatar diversity on user engagement (Ferraro et al., 2024). Results also provide initial support for how diversity perceptions change with time and the communities using avatars (Shemla et al., 2024). Consequently, we illustrate a means to capture the multidimensionality of perceived avatar diversity beyond a problematic focus on infinite human attribute differences.

Further, we support Shemla et al. (2024) in challenging the conflation of perceived diversity with objective diversity. Based on our model, perceived diversity extends beyond visible differences to encompass shifting perceptions and the affective responses to representation using artificial entities. The variable outcomes in convergent and discriminant validity across our three ADPS dimensions reflect their varying degrees of distinctiveness with other constructs and uncover the potentially suppressed effects of avatar diversity. While positive outcomes could result from a perception of heterogeneity, negative outcomes could still arise if the diversity concerns are not addressed. Future theoretical work is essential to establish how the ADPS dimensions influence downstream outcomes differentially.

Expanding on the previous point, we did not find support for the expected correlations between the perceived heterogeneity of the ADPS and pro-diversity beliefs, openness to experience, and technology acceptance. We also did not find support between diversity concerns of the ADPS and perceived diversity, pro-diversity beliefs, and technology acceptance. Our findings suggest that the perceived heterogeneity and diversity concerns related to avatars are intertwined more with its technology than with factors influencing perceived human diversity. Separately, the strong validity of context-dependent diversity, which aligns with all predictions from existing literature (Ashton and Lee, 2009; Homan et al., 2010; Homan et al., 2007), suggests it is the dimension most aligned with how people perceive actual human diversity. Collectively, these indicate that predictions based on human diversity cannot be directly applied to the avatar context. The ADPS with its three dimensions illuminate where there are overlaps between avatar diversity and human diversity, respectively.

Critically, our findings on diversity concerns reveal that the negative affective reactions are consequences of the perceptibility, authenticity, and relatability of avatars, rather than identity or stereotype threats that are established in diversity literature (Stephan et al., 2015; Tajfel et al., 1979; Tajfel and Turner, 2004). Such adverse reactions or “bad representations” are driven by design and representation failures, rather than an us-vs.-them divide and intergroup bias. This means that resolving diversity issues in the avatar context necessitates not only anti-bias training but also improving design processes. It also implies that theories on the psychological dynamics surrounding the unique characteristics of human avatars, such as the uncanny valley theory (Mori, 1970), may have additional explanatory power than social perception theories alone in understanding diversity in virtual contexts. Broadly, our study supports the need for integrative theoretical perspectives on perceived avatar diversity. Future experimental studies that manipulate avatar customization features are needed to test if diversity concerns track with technology affordances. Such a design could compare users of a system with extensive vs. limited customization options, isolating the effect of technology from users’ preexisting beliefs about human diversity.

Finally, our study presents limitations that open avenues for future theoretical development. By implication of our methodological approach, we have adopted an abstracted frame of diversity and thus constrained users’ diversity perceptions in like manner. This likely accounts for the relatively modest variance explained by our ADPS items. Human diversity studies suggest that people construe diversity from a concrete/proximal or abstract/distal perspective (Jaffé et al., 2019; Toma et al., 2025). Since our study could not have assessed both simultaneously, future research is essential to illuminate perceptions of avatar diversity when it is psychologically near, such as by assessing how users perceive avatar diversity when actively participating in a specific avatar-mediated platform. This should provide an even more holistic theorization of diversity perceptions in the avatar context.

### 7.2 Practical implications

This study offers significant practical implications for both academic research and industry practice. For researchers, our study presents an initial model and measure to examine perceived avatar diversity, including hypothesizing potential antecedents and specifying distinct psychological mechanisms that connect each ADPS dimension with user engagement, advertising, or other related outcomes. For practitioners, our findings provide actionable guidance to create more inclusive avatar-mediated environments. Avatar creation tools and processes need to be simultaneously heterogeneous, authentic, relatable, and dynamic for diversity perceptions to be favorable, thereby enhancing user satisfaction and achieving commercial goals.

### 7.3 Limitations and future directions

Study limitations are typical of early theory building. Variance explained in EFA is modest, and some items moved across our proposed dimensions. Moreover, the CFA model required adjustments before it was stabilized. Thus, any claims about precise dimensional boundaries or mean-level comparisons should be made with caution. We present our findings as an initial conceptual map for perceived avatar diversity, rather than a finalized model and measure.

Our study did not find consistent support for salience and representation fidelity as distinct dimensions of perceived diversity. The challenge with salience could be due to a methodological artifact of self-rated measurements. Specifically, the very act of asking participants to evaluate avatar diversity necessarily makes it salient. Future studies could overcome this by employing psychophysical approaches like eye-tracking to capture visual attention. Besides, while the inconsistent support for fidelity could be attributed to statistical noise, our findings on diversity concerns suggest that representation fidelity may have collapsed with the sense of representation to reflect a single, broader construct of authentic and relatable representation. Users do not seem to separate the technical accuracy of a representation from feelings of being seen. Future studies should aim to test a revised model where these elements are facets of such a broader construct, rather than independent dimensions.

## Data Availability

The raw data supporting the conclusions of this article will be made available by the authors, without undue reservation.

## References

[ref1] Abrams D. Thomas J. Hogg M. A. (1990). Numerical distinctiveness, social identity and gender salience. Br. J. Soc. Psychol. 29, 87–92.

[ref2] Appel M. Weber S. (2021). Do mass mediated stereotypes harm members of negatively stereotyped groups? A meta-analytical review on media-generated stereotype threat and stereotype lift. Commun. Res. 48, 151–179. doi: 10.1177/0093650217715543

[ref3] Ashton M. C. Lee K. (2009). The HEXACO–60: a short measure of the major dimensions of personality. J. Pers. Assess. 91, 340–345. doi: 10.1080/00223890902935878, PMID: 20017063

[ref4] Bailenson J. N. Beall A. C. (2006). “Transformed social interaction: exploring the digital plasticity of avatars” in Avatars at work and play: Collaboration and interaction in shared virtual environments. eds. Schroeder R. Axelsson A.-S. (Dordrecht: Springer), 1–16.

[ref5] Bangor A. Kortum P. T. Miller J. T. (2008). An empirical evaluation of the system usability scale. Int. J. Hum.-Comput. Interact. 24, 574–594. doi: 10.1080/10447310802205776

[ref6] Baumeister R. F. Leary M. R. (2017). “The need to belong: desire for interpersonal attachments as a fundamental human motivation” in Interpersonal development. eds. Baumeister R. F. Leary M. R. (Abingdon, Oxfordshire: Routledge), 57–89.

[ref7] Brooke J. (1996). “SUS-A quick and dirty usability scale” in Usability evaluation in industry. eds. Jordan P. W. Thomas B. McClelland I. L. Weerdmeester B. (London: CRC Press), 4–7.

[ref8] Burden D. Savin-Baden M. (2019). Virtual humans: Today and tomorrow. New York: Chapman and Hall/CRC.

[ref9] Burgess A. J. Wilkie D. C. H. Dolan R. (2021). Towards successful diversity initiatives: the importance of building audience connectedness. J. Mark. Manag. 37, 144–161. doi: 10.1080/0267257X.2020.1844278, PMID: 40989069

[ref10] Burgess A. Wilkie D. C. H. Dolan R. (2024). The power of beliefs: how diversity advertising builds audience connectedness. Eur. J. Mark. 58, 1969–1994. doi: 10.1108/EJM-01-2023-0051

[ref11] Campbell C. Sands S. McFerran B. Mavrommatis A. (2025). Diversity representation in advertising. J. Acad. Mark. Sci. 53, 588–616. doi: 10.1007/s11747-023-00994-8

[ref12] Child D. (2006). The essentials of factor analysis. London and New York: A&C Black.

[ref13] Cohen C. E. (1981). Person categories and social perception: testing some boundaries of the processing effect of prior knowledge. J. Pers. Soc. Psychol. 40, 441–452.

[ref14] Connor P. Weeks M. Glaser J. Chen S. Keltner D. (2022). Intersectional implicit bias: evidence for asymmetrically compounding bias and the predominance of target gender. J. Pers. Soc. Psychol. 124, 22–48. doi: 10.1037/pspa0000314 35587425

[ref15] Daniels D. P. Neale M. A. Greer L. L. (2017). Spillover bias in diversity judgment. Organ. Behav. Hum. Decis. Process. 139, 92–105. doi: 10.1016/j.obhdp.2016.12.005

[ref16] Do T. D. Zelenty S. Gonzalez-Franco M. McMahan R. P. (2023). Valid: a perceptually validated virtual avatar library for inclusion and diversity. Front. Virtual Real. 4:1248915. doi: 10.3389/frvir.2023.1248915

[ref17] Eisend M. Muldrow A. F. Rosengren S. (2023). Diversity and inclusion in advertising research. Int. J. Advert. 42, 52–59. doi: 10.1080/02650487.2022.2122252

[ref18] Fellnhofer K. Puumalainen K. Sjögrén H. (2017). Entrepreneurial orientation in work groups – effects of individuals and group characteristics. Int. Entrep. Manag. J. 13, 427–463. doi: 10.1007/s11365-016-0408-5

[ref19] Ferraro C. Sands S. Zubcevic-Basic N. Campbell C. (2024). Diversity in the digital age: how consumers respond to diverse virtual influencers. Int. J. Advert., 43, 1342–1365. doi: 10.1080/02650487.2023.2300927

[ref20] Ge L. Yip W. F. Li R. Chua E. S. S. Ho M.-H. R. Ho A. H. Y. . (2025). Development and validation of the multi-dimensional health resilience scale for community-dwelling adults. Front. Public Health 13:1452738. doi: 10.3389/fpubh.2025.1452738 40013045 PMC11864133

[ref21] Goffman E. (1959). The presentation of self in everyday life. New York: Doubleday.

[ref22] Hair J. F. Black W. C. Babin B. J. Anderson R. E. (2019). Multivariate data analysis. Andover, Hampshire: Cengage.

[ref23] Han S. Pistole M. C. (2017). Big five personality factors and facets as predictors of openness to diversity. Aust. J. Psychol. 151, 752–766. doi: 10.1080/00223980.2017.1393377, PMID: 29166225

[ref24] Harrison D. A. Price K. H. Bell M. P. (1998). Beyond relational demography: time and the effects of surface-and deep-level diversity on work group cohesion. Acad. Manag. J. 41, 96–107.

[ref25] Harrison D. A. Price K. H. Gavin J. H. Florey A. T. (2002). Time, teams, and task performance: changing effects of surface-and deep-level diversity on group functioning. Acad. Manag. J. 45, 1029–1045. doi: 10.5465/3069328

[ref26] Homan A. C. Greer L. L. Jehn K. A. Koning L. (2010). Believing shapes seeing: the impact of diversity beliefs on the construal of group composition. Group Process. Intergroup Relat. 13, 477–493. doi: 10.1177/1368430209350747

[ref27] Homan A. C. Knippenberg D. v. Kleef G. A. V. Dreu C. K. W. D. (2007). Bridging faultlines by valuing diversity: diversity beliefs, information elaboration, and performance in diverse work groups. J. Appl. Psychol. 92, 1189–1199. doi: 10.1037/0021-9010.92.5.1189, PMID: 17845079

[ref28] Hong J.-W. Cruz I. F. Kim D. (2024). Justice behind the virtual mask: the influence of race of the virtual influencer and the creator on promoting the Black lives matter movement. New Media Soc. doi: 10.1177/14614448241262806

[ref29] Iyer A. (2022). Understanding advantaged groups' opposition to diversity, equity, and inclusion (DEI) policies: the role of perceived threat. Soc. Personal. Psychol. Compass 16:e12666. doi: 10.1111/spc3.12666

[ref30] Jaffé M. E. Jeitziner L. Keller M. D. Walker M. (2022). Differences in faces do make a difference: diversity perceptions and preferences in faces. J. Exp. Soc. Psychol. 100. doi: 10.1016/j.jesp.2021.104277

[ref31] Jaffé M. E. Rudert S. C. Greifeneder R. (2019). You should go for diversity, but I'd rather stay with similar others: social distance modulates the preference for diversity. J. Exp. Soc. Psychol. 85:103881. doi: 10.1016/j.jesp.2019.103881

[ref32] Jiang K. (2023). From science fiction to reality: How digital humans are forging new realities. Forbes. Available online at: https://www.forbes.com/councils/forbestechcouncil/2023/06/08/from-science-fiction-to-reality-how-digital-humans-are-forging-new-realities/ (Accessed July 05, 2025).

[ref33] Kauff M. Stegmann S. Van Dick R. Beierlein C. Christ O. (2019). Measuring beliefs in the instrumentality of ethnic diversity: development and validation of the pro-diversity beliefs scale (PDBS). Group Process. Intergroup Relat. 22, 494–510. doi: 10.1177/1368430218767025

[ref34] Kim I. Ki C.-W. Lee H. Kim Y.-K. (2024). Virtual influencer marketing: evaluating the influence of virtual influencers’ form realism and behavioral realism on consumer ambivalence and marketing performance. J. Bus. Res. 176:114611. doi: 10.1016/j.jbusres.2024.114611

[ref35] Kratz J. (2024). The little known history of DEI and why it’s critical to its survival. Forbes.

[ref36] Lee J.-E. R. (2014). Does virtual diversity matter?: effects of avatar-based diversity representation on willingness to express offline racial identity and avatar customization. Comput. Hum. Behav. 36, 190–197. doi: 10.1016/j.chb.2014.03.040

[ref37] Lee Y.-H. Xiao M. Wells R. H. (2018). The effects of avatars' age on older adults' self-disclosure and trust. Cyberpsychol. Behav. Soc. Netw. 21, 173–178. doi: 10.1089/cyber.2017.0451, PMID: 29638156

[ref38] Lee Y.-H. Yuan C. W. T. (2023). I’m not a puppet, I’ma real boy! Gender presentations by virtual influencers and how they are received. Comput. Hum. Behav. 149:107927. doi: 10.1016/j.chb.2023.107927

[ref39] Lehdonvirta M. Nagashima Y. Lehdonvirta V. Baba A. (2012). The stoic male: how avatar gender affects help-seeking behavior in an online game. Games Cult. 7, 29–47. doi: 10.1177/1555412012440307

[ref40] Lindsey A. King E. Hebl M. Levine N. (2015). The impact of method, motivation, and empathy on diversity training effectiveness. J. Bus. Psychol. 30, 605–619. doi: 10.1007/s10869-014-9384-3

[ref41] Mack K. Hsu R. C. L. Monroy-Hernández A. Smith B. A. Liu F. (2023). “Towards inclusive avatars: disability representation in avatar platforms.” *Proceedings of the 2023 CHI Conference on Human Factors in Computing Systems*, 1–13.

[ref42] Mayo M. van Knippenberg D. Guillén L. Firfiray S. (2016). Team diversity and categorization salience: capturing diversity-blind, intergroup-biased, and multicultural perceptions. Organ. Res. Methods 19, 433–474. doi: 10.1177/1094428116639130

[ref43] Messinger P. R. Ge X. Smirnov K. Stroulia E. Lyons K. (2019). Reflections of the extended self: visual self-representation in avatar-mediated environments. J. Bus. Res. 100, 531–546. doi: 10.1016/j.jbusres.2018.12.020

[ref44] Meyer B. Shemla M. Schermuly C. C. (2011). Social category salience moderates the effect of diversity faultlines on information elaboration. Small Group Res. 42, 257–282. doi: 10.1177/1046496411398396

[ref45] Miao F. Kozlenkova I. V. Wang H. Xie T. Palmatier R. W. (2022). An emerging theory of avatar marketing. J. Mark. 86, 67–90. doi: 10.1177/0022242921996646

[ref46] Morgan H. Donovan A. Almeida R. Lin A. Perry Y. (2020). The role of the avatar in gaming for trans and gender diverse young people. Int. J. Environ. Res. Public Health 17:8617. doi: 10.3390/ijerph17228617 33233536 PMC7699515

[ref47] Mori M. (1970). Bukimi no tani [the uncanny valley]. Energy 7:33.

[ref48] Nowak K. L. (2004). The influence of anthropomorphism and agency on social judgment in virtual environments. J. Comput.-Mediat. Commun. 9:JCMC925. doi: 10.1111/j.1083-6101.2004.tb00284.x

[ref49] Nowak K. L. Rauh C. (2005). The influence of the avatar on online perceptions of anthropomorphism, androgyny, credibility, homophily, and attraction. J. Comput.-Mediat. Commun. 11, 153–178. doi: 10.1111/j.1083-6101.2006.tb00308.x

[ref50] Petsko C. D. Rosette A. S. Bodenhausen G. V. (2022). Through the looking glass: a lens-based account of intersectional stereotyping. J. Pers. Soc. Psychol. 123, 763–787. doi: 10.1037/pspi0000382, PMID: 35025602

[ref51] Phillips K. W. Loyd D. L. (2006). When surface and deep-level diversity collide: the effects on dissenting group members. Organ. Behav. Hum. Decis. Process. 99, 143–160. doi: 10.1016/j.obhdp.2005.12.001

[ref52] Phillips K. W. Northcraft G. B. Neale M. A. (2006). Surface-level diversity and decision-making in groups: when does deep-level similarity help? Group Process. Intergroup Relat. 9, 467–482. doi: 10.1177/1368430206067557

[ref53] Ramasubramanian S. Riewestahl E. Ramirez A. (2023). “Race and ethnic stereotypes in the media” in Oxford research Encyclopedia of communication. doi: 10.1093/acrefore/9780190228613.013.1262

[ref54] Randel A. E. (2002). Identity salience: a moderator of the relationship between group gender composition and work group conflict. J. Organ. Behav. 23, 749–766. doi: 10.1002/job.163

[ref55] Sands S. Demsar V. Ferraro C. Campbell C. Cohen J. (2024). Inauthentic inclusion: exploring how intention to use AI-generated diverse models can backfire. Psychol. Mark. 41, 1396–1413. doi: 10.1002/mar.21987

[ref56] Santoniccolo F. Trombetta T. Paradiso M. N. Rollè L. (2023). Gender and media representations: a review of the literature on gender stereotypes, objectification and sexualization. Int. J. Environ. Res. Public Health 20:5770. doi: 10.3390/ijerph20105770, PMID: 37239498 PMC10218532

[ref57] Schober P. Boer C. Schwarte L. A. (2018). Correlation coefficients: appropriate use and interpretation. Anesth. Analg. 126, 1763–1768. doi: 10.1213/ANE.0000000000002864, PMID: 29481436

[ref58] Shemla M. Meyer B. Greer L. Jehn K. A. (2016). A review of perceived diversity in teams: does how members perceive their team's composition affect team processes and outcomes? J. Organ. Behav. 37, S89–S106. doi: 10.1002/job.1957

[ref59] Shemla M. Meyer B. Grgic J. (2024). Perceived diversity in teams: conceptualizations, effects, and new research avenues. Curr. Opin. Psychol. 60:101925. doi: 10.1016/j.copsyc.2024.101925, PMID: 39395356

[ref60] Starck J. G. Sinclair S. Shelton J. N. (2021). How university diversity rationales inform student preferences and outcomes. Proc. Natl. Acad. Sci. 118:e2013833118. doi: 10.1073/pnas.2013833118, PMID: 33846243 PMC8072243

[ref61] Stephan W. G. Ybarra O. Rios K. (2015). “Intergroup threat theory” in Handbook of prejudice, stereotyping, and discrimination. ed. Nelson T. D. (New York: Psychology Press), 255–278.

[ref62] Tajfel H. Turner J. C. (2004). “The social identity theory of intergroup behavior” in Political psychology: Key readings. eds. Jost J. T. Sidanius J. (New York: Psychology Press), 276–293.

[ref63] Tajfel H. Turner J. C. Austin W. G. Worchel S. (1979). “An integrative theory of intergroup conflict” in Organizational identity: A reader. eds. Hatch M. J. Schultz M. (Oxford: Oxford University Press), 56–65.

[ref64] Tessema M. T. Hulback T. Jones J. Santos-Leslie R. Ninham K. Sterbin A. . (2023). Diversity, equity, and inclusion: history, climate, benefits, challenges, and creative strategies. J. Hum. Resour. Sustain. Stud. 11, 780–794. doi: 10.4236/jhrss.2023.114044

[ref65] Toma C. Carter A. B. Phillips K. W. (2025). Diversity? Great for most just less so for me: how cognitive abstraction affects diversity attitudes and choices. Behav. Sci. 15:585. doi: 10.3390/bs15050585, PMID: 40426363 PMC12109399

[ref66] Unzueta M. M. Knowles E. D. Ho G. C. (2012). Diversity is what you want it to be: how social-dominance motives affect construals of diversity. Psychol. Sci. 23, 303–309. doi: 10.1177/0956797611426727, PMID: 22368153

[ref67] van Knippenberg D. De Dreu C. K. W. Homan A. C. (2004). Work group diversity and group performance: an integrative model and research agenda. J. Appl. Psychol. 89, 1008–1022. doi: 10.1037/0021-9010.89.6.1008, PMID: 15584838

[ref68] van Knippenberg D. Haslam S. A. Platow M. J. (2007). Unity through diversity: value-in-diversity beliefs, work group diversity, and group identification. Group Dyn. Theory Res. Pract. 11, 207–222. doi: 10.1037/1089-2699.11.3.207

[ref69] Waltemate T. Gall D. Roth D. Botsch M. Latoschik M. E. (2018). The impact of avatar personalization and immersion on virtual body ownership, presence, and emotional response. IEEE Trans. Vis. Comput. Graph. 24, 1643–1652. doi: 10.1109/TVCG.2018.2794629, PMID: 29543180

[ref70] Walther J. B. (2007). Selective self-presentation in computer-mediated communication: hyperpersonal dimensions of technology, language, and cognition. Comput. Hum. Behav. 23, 2538–2557. doi: 10.1016/j.chb.2006.05.002

[ref71] Walther J. B. Lew Z. (2022). Self-transformation online through alternative presentations of self: a review, critique, and call for research. Ann. Int. Commun. Assoc. 46, 135–158. doi: 10.1080/23808985.2022.2096662, PMID: 40989069

[ref72] Williams K. O’Reilly C. (1998). Forty years of diversity research: a review. Res. Organ. Behav. 20, 77–140.

[ref73] Yang G. S. Gibson B. Lueke A. K. Huesmann L. R. Bushman B. J. (2014). Effects of avatar race in violent video games on racial attitudes and aggression. Soc. Psychol. Personal. Sci. 5, 698–704. doi: 10.1177/1948550614528008

[ref74] Zhang Y. G. Dang Y. M. Brown S. A. Chen H. (2017). Investigating the impacts of avatar gender, avatar age, and region theme on avatar physical activity in the virtual world. Comput. Hum. Behav. 68, 378–387. doi: 10.1016/j.chb.2016.11.052

[ref75] Zhang Y. G. Dang M. Y. Chen H. (2020). An explorative study on the virtual world: investigating the avatar gender and avatar age differences in their social interactions for help-seeking. Inf. Syst. Front. 22, 911–925. doi: 10.1007/s10796-019-09904-2

[ref76] Zhang K. Deldari E. Lu Z. Yao Y. Zhao Y. (2022), “It’s just part of me: understanding avatar diversity and self-presentation of people with disabilities in social virtual reality.” Proceedings of the 24th International ACM SIGACCESS Conference on Computers and Accessibility, 1–16.

