# Supplementary material for: Avatar diversity perception scale (ADPS): a new multidimensional measure for perceived human avatar diversity
Source: Front Psychol. 2025 Nov 11;16:1705591. doi: 10.3389/fpsyg.2025.1705591 (PMC12644037; doi:10.3389/fpsyg.2025.1705591)
Supplement: Supplementary file 1 [file Supplementary_file_1.docx]

**Appendix A**

*Original 65-item Avatar Diversity Perception Scale*

| Based on your experience most of the time, rate the extent to which you agree with the following statements regarding the diversity of human avatars. | | |
| --- | --- | --- |
| Proposed dimension | Item no. | Item description |
| Perceived heterogeneity | 1 | There is variation in general. |
|  | 2 | There is difference in the looks. |
|  | 3 | There are different expressions on the face. |
|  | 4 | There is difference in actions. |
|  | 5 | There is representation of various groups, identities, and perspectives. |
|  | 6 | There is very few variety. |
|  | 7 | There is a good representation of various interest groups and communities. |
|  | 8 | There are different skin colors, facial features, hair, body shapes and physical disabilities. |
|  | 9 | There is not just one uniform look. |
|  | 10 | It is unlikely that any two avatars are exactly the same. |
|  | 11 | It is all the same. |
|  | 12 | There is a range of different features. |
|  | 13 | Things are as different as they can be. |
| Salience | 14 | I do not really notice the diversity. |
|  | 15 | Certain personality traits cannot be portrayed easily. |
|  | 16 | In some ways diversity is not so immediately discernible. |
|  | 17 | The most obvious one is difference in skin tones. |
|  | 18 | It is obvious that there is already diversity. |
|  | 19 | The more people create different avatars, the more visible the diversity. |
|  | 20 | People can show their inner self. |
|  | 21 | Avatars without a full body make diversity less visible. |
|  | 22 | It is hard to spot distinct differences. |
|  | 23 | I am aware of diversity when I have something in mind but the platform does not have that. |
|  | 24 | Diversity is very visible when you are the minority. |
|  | 25 | If everyone is using more or less a standardized character, then diversity will not be that obvious. |
|  | 26 | If you are the majority, diversity is not something you notice very much. |
| Sense of representation | 27 | I feel like I am not represented. |
|  | 28 | A lot of the minority do not get a chance to see themselves being represented. |
|  | 29 | I know how it feels like not to see avatars who look like me. |
|  | 30 | I would not want to be pressured to pursue a certain look. |
|  | 31 | Not everyone can relate to what is represented. |
|  | 32 | To someone else, I question if there is anything offensive. |
|  | 33 | I might have the same avatar combination as another guy, even though we are not the same person in real life. |
|  | 34 | I cannot even relate to what is represented. |
|  | 35 | Diversity is a sensitive topic. |
|  | 36 | I am not the target audience. |
|  | 37 | The people that are creating avatars need to be diverse in order for avatars to be diverse. |
|  | 38 | The diversity makes people feel uncomfortable. |
|  | 39 | People want to be included, whether it is in real life or the digital world. |
| Representation fidelity | 40 | The avatars do not even look human. |
|  | 41 | There are no in-betweens. |
|  | 42 | The features are still not the closest representation of different people. |
|  | 43 | Certain things (e.g., features) can be improved. |
|  | 44 | Diversity is implemented in a way that is very stereotyping. |
|  | 45 | I question if the diversity is real. |
|  | 46 | If the tool is limited, then the feature is limited. |
|  | 47 | The diversity is neutral and non-offensive. |
|  | 48 | Many sites do not verify your identity. |
|  | 49 | At the end of the day, the diversity is manmade. |
|  | 50 | No one can ever be what is represented. |
|  | 51 | You do not get to see the actual person behind it. |
|  | 52 | I do not know if people have the kind of resources to find out and design all the unique human features. |
| Context-dependent diversity | 53 | Diversity depends on the context. |
|  | 54 | Diversity is work in progress. |
|  | 55 | Diversity can be achieved over time. |
|  | 56 | Diversity is anything that is rooted in the community. |
|  | 57 | Diversity is anything that shapes the community. |
|  | 58 | Diversity might not be possible in some cases. |
|  | 59 | Diversity is evolving all the time. |
|  | 60 | There is a kind of diversity that I cannot accept. |
|  | 61 | Diversity depends on what the developers want to include in their character creation tools. |
|  | 62 | I hear about certain aspect(s) of diversity a lot in the news. |
|  | 63 | There are certain types of diversity with greater impact. |
|  | 64 | Diversity is politically volatile. |
|  | 65 | Society is still trying to accept this. |
